# A questionnaire survey of radiological diagnosis and management of renal dysplasia in children

**DOI:** 10.1007/s40620-017-0417-7

**Published:** 2017-06-24

**Authors:** Giovanni Montini, Marco Busutti, Fatos Yalcinkaya, Adrian S. Woolf, Stefanie Weber, On behalf of the European Society for Paediatric Nephrology Working Group on Congenital Anomalies of the Kidney and Urinary Tract, Harika Alpay, Gema Ariceta, An Bael, Marjolein Bonthuis, Salim Caliskan, Elene Gotsiridze, Kaan Gulleroglu, Zubeyde Gunduz, Duygu Ovunc Hacihamdioglu, Julia Hoefele, Khalid Ismaili, Helena Jardim, Silvio Maringhini, Giovanni Montini, Luisa Murer, Birsin Ozcakar, Paloma Parvex, Svetlana Paunova, Amira Peco Antic, Ann Raes, Irakli Rtskhiladze, Thierry Schurmans, Fatma Lale Sever, Velibor Tasic, Kjell Tullus, Albertien Van Eerde, Stefanie Weber, Adrian S. Woolf, Fatos Yalcinkaya, Nurdan Yildiz, Alev Yilmaz, Selcuk Yuksel, Marcin Zaniew

**Affiliations:** 1 Pediatric Nephrology and Dialysis Unit, Department of Clinical Sciences and Community Health, University of Milan, Fondazione IRCCS Cà Granda-Ospedale Maggiore Policlinico, Via della Commenda 9, 20122 Milano, Italy; 2 Division of Nephrology, Dialysis and Transplant, Dipartimento Chirurgico, Medico, Odontoiatrico e di Scienze Morfologiche con Interesse Trapiantologico, Oncologico e di Medicina Rigenerativa, Azienda Ospedaliero-Universitaria Policlinico di Modena, University of Modena and Reggio Emilia, Modena, Italy; 3 Department of Pediatric Nephrology, Ankara University Faculty of Medicine, Ankara, Turkey; 4 School of Biological Sciences, Faculty of Biology Medicine and Health, University of Manchester, Manchester, UK; 5 Department of Pediatric Nephrology, University Children’s Hospital Marburg, Philipps-University Marburg, Marbug, Germany

**Keywords:** Renal dysplasia, Chronic kidney disease, CAKUT, Children, Ultrasonography, Genetic testing

## Abstract

**Background:**

The condition called renal dysplasia is considered to be a frequent cause of chronic kidney disease in children. Formally, it is defined by histological parameters. In current nephrology practice, however, the appearance of the kidneys on ultrasound scanning is often used as a basis for the diagnosis.

**Methods:**

The European Society for Pediatric Nephrology Working Group on Congenital Anomalies of the Kidney and Urinary Tract hypothesized that the current diagnostic approach with regard to renal dysplasia was not homogeneous. Accordingly, we here report the results of a survey targeting pediatric nephrologists with 12 questions regarding their perceptions of the ultrasonographic characteristics of renal dysplasia and further tests that they might undertake.

**Results:**

Of almost 1200 physicians who successfully received the invitation, 248 from 54 countries completed the survey. There was a notable lack of homogeneity regarding the ultrasonographic diagnosis of renal dysplasia and also of follow-up tests, including genetic testing and further radiology.

**Conclusions:**

Based on the responses to this large survey, a picture emerges of nephrologists’ current clinical practice with regard to renal dysplasia. The Working Group considers that these results serve as an important sounding board which can provide the basis for more definitive recommendations regarding the challenges to clinical diagnosis and diagnostic follow-up of this important condition.

## Introduction

The Miller-Keane Encyclopedia and Dictionary of Medicine defines dysplasia as an abnormal tissue development caused by alteration in the size, shape and organization of mature cells [1]. In particular, renal dysplasia (RD), which can be either uni- or bilateral, is a congenital disorder, characterized by abnormal differentiation in the renal parenchyma, histologically defined by the presence of primitive ducts and nests of metaplastic cartilage [2, 3]. Renal hypoplasia is conversely characterized by a reduced number of nephrons, in a small kidney (below −2SD), with normal renal architecture. Formally, RD is defined by histological parameters. However, to perform a kidney biopsy in every suspected case of RD is not feasible. Thus, in everyday nephrology practice, imaging plays a major role in the diagnosis of these malformations.

Renal dysplasia is part of the spectrum of congenital anomalies of the kidney and urinary tract (CAKUT). It can occur isolated or as part of a syndrome. Isolated CAKUT can run in families. The clinical picture incorporates a wide and varied spectrum of manifestations, depending on the number of kidneys involved, the severity of dysplasia, the time at diagnosis and the presence or absence of oligohydramnios. Unilateral forms (incidence 1:1000–5000) are usually asymptomatic and incidentally diagnosed during abdominal pre- and postnatal ultrasonography [4]; patients with bilateral forms (incidence 1:5000–10,000) are at risk of decreased renal function and end-stage renal disease and these forms are often associated with urologic malformations, such as dilating vesicoureteral reflux or urethral valves. Renal dysplasia is considered to represent the most common cause of chronic kidney disease (CKD) in pediatric registries [5, 6], and children with RD are frequently the object of trials, the outcomes of which carry potential clinical implications for this population [7].

Despite the fact that most cases of RD do not appear to be familial, mutations in several genes such as *TCF2*/*HNF1B* (mostly), *PAX2, EYA1, SIX1* and *SALL1* have been identified. For example, the ESCAPE consortium evaluated the prevalence of gene mutations in children with overtly non-syndromic RD and detected alterations in 16% of index cases [8]. These genes encode for transcription factors and related molecules critical for early renal development, and their mutations are sometimes associated with complex clinical syndromes.

The European Society for Pediatric Nephrology (ESPN) Working Group on Congenital Anomalies of the Kidney and Urinary Tract hypothesized that the current diagnostic approach with regard to renal dysplasia was not homogeneous. We were also mindful of the observation that the appearance of kidneys on ultrasound (US) scanning is often currently used as a basis for the diagnosis of RD. Accordingly, we here report the results of a survey targeting pediatric nephrologists with 12 questions regarding their perceptions of the ultrasonographic characteristics of renal dysplasia and further tests that they might undertake.

## Materials and methods

The survey was conducted on the Survey Monkey platform (https://www.surveymonkey.com) in the form of a questionnaire with 12 questions regarding the definition, diagnosis, imaging and genetic testing of RD. A letter explaining the questionnaire and the rationale behind it was sent to the mailing list of the ESPN on two different dates between 2014 and 2015. The questions and possible answers are listed in the following tables. They cover three principal topics: Table 1: diagnosis (questions 1–3); Table 2: management (questions 4–7); Table 3: genetics (questions 8–12).

**Table 1:** Definition and diagnosis: questions 1–3

1. Is the presence of small kidneys necessary for the diagnosis of dysplasia?
a. Yes
b. No
2. Is the presence of hyperechogenicity necessary for the diagnosis of dysplasia?
a. Yes
b. No
3. Which of the following definitions represent a typical diagnosis of renal dysplasia in your opinion? (more than one answer is possible)
a. Small bilateral kidneys
b. Small unilateral kidney
c. Increased renal parenchymal echogenicity in a small kidney
d. Increased renal parenchymal echogenicity in a big kidney
e. Increased renal parenchymal echogenicity in kidneys with cysts
f. Other

**Table 2:** Imaging: questions 4–7

4. When would you recommend further imaging in children with a diagnosis of renal dysplasia within the first year of life? (more than one answer is possible)
a. Both kidneys affected
b. Both kidneys affected and presence of oligohydramnios
c. One kidney affected in association with dilation of the urinary tract
d. Both kidneys affected and abnormal renal function
e. One very small kidney affected with no dilation of the urinary tract
5. What further imaging would you suggest? (more than one answer is possible)
a. Voiding cystourethrography
b. DMSA scan
c. MRI
d. CT scan
6. In a child with bilateral renal dysplasia would you suggest sonographic screening of the first degree relatives?
a. Yes
b. No
7. In a child with unilateral renal dysplasia would you suggest sonographic screening of the first degree relatives?
a. Yes
b. No

**Table 3:** Genetics: questions 8–12

8. In a child with unilateral renal dysplasia would you suggest genetic screening?
a. Yes
b. No
9. In a child with bilateral renal dysplasia would you suggest genetic screening?
a. Yes
b. No
10. In a child with unilateral cystic renal dysplasia would you suggest genetic screening?
a. Yes
b. No
11. In a family with bilateral cystic renal dysplasia would you suggest genetic screening?
a. Yes
b. No
12. Which genes would you screen in a family with bilateral renal dysplasia and other urologic anomalies and or extra-renal manifestations?
a. HNF1β
b. PAX2
c. EYA1
d. SIX1
e. Whole exome sequencing
f. Other

## Results

Of almost 1200 physicians who successfully received the invitation, 248 from 54 countries completed the survey. Most of them were Pediatric Nephrologists (87%) from European countries, in particular: 51 from Turkey, 25 from Italy, 19 from the UK, 17 from Germany.

### Definition and diagnosis

**Fig. 1:**
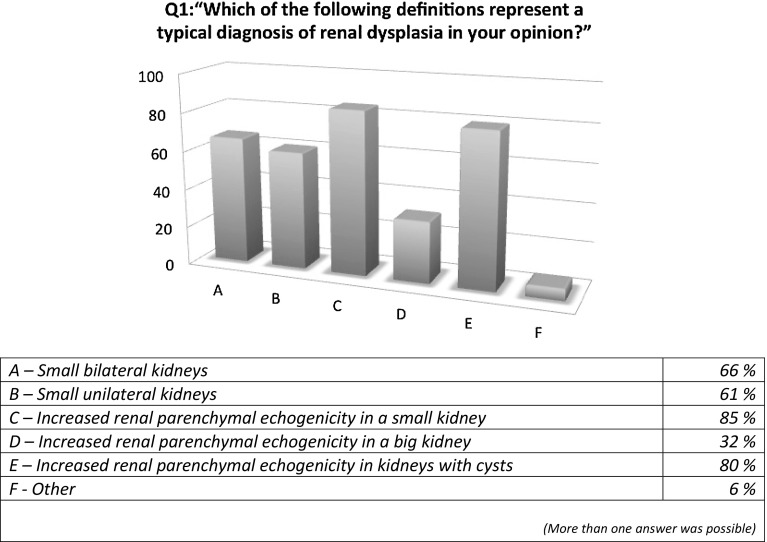
Definition of renal dysplasia

The responders’ definition of dysplasia is shown in Fig. 1. More than 1 answer was possible for this question. While an increased parenchymal echogenicity in a small kidney represents the most commonly selected option, followed by increased parenchymal echogenicity in kidneys with cysts, more than 60% of responders declared that small kidneys (unilateral or bilateral) were a typical sign of RD. This tendency was also confirmed in question 2, where almost a quarter of responders affirmed that the finding of small kidneys was necessary for a diagnosis of RD. In question 3, 58% of responders answered that the presence of hyperechogenicity was not necessary for the diagnosis of dysplasia.

### Imaging

**Fig. 2:**
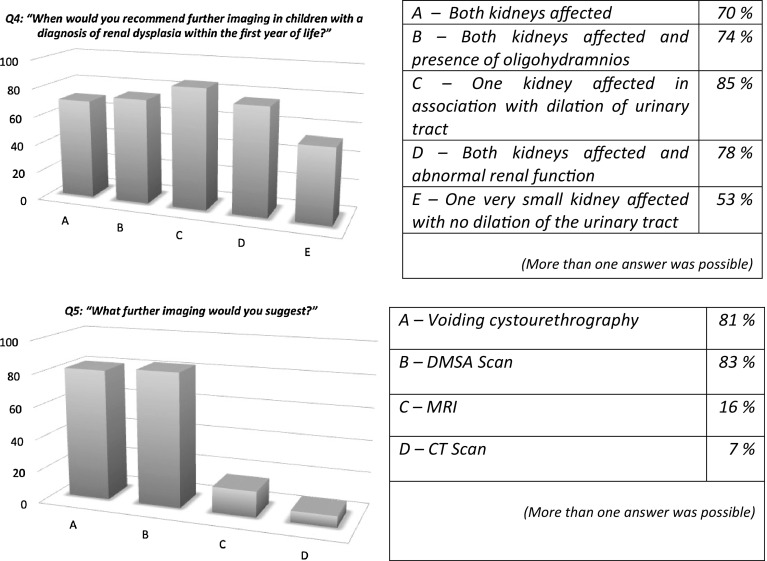
Imaging of renal dysplasia

As we can see from the responses to question 4 (Fig. 2), most physicians agreed on recommending further imaging in the majority of cases, with the highest percentage prescribing further imaging in children with urinary tract dilation. The presence of oligohydramnios was also considered a risk factor requiring further imaging, as it is suggestive of posterior urethral valves in males. The preferred imaging exams were voiding cystourethrography (VCUG) and dimercaptosuccinic acid (DMSA) scanning, while a minority of responders preferred to perform magnetic resonance imaging (MRI) or a computed tomography (CT) scan (Fig. 2).

Due to the documented familial occurrence of RD, we raised the question of imaging screening for the relatives of affected children. The responders were more likely to perform US screening in relatives of children affected by bilateral RD (over 70%) than in unilateral RD (less than 40%) (questions 6 and 7).

### Genetics

**Fig. 3:**
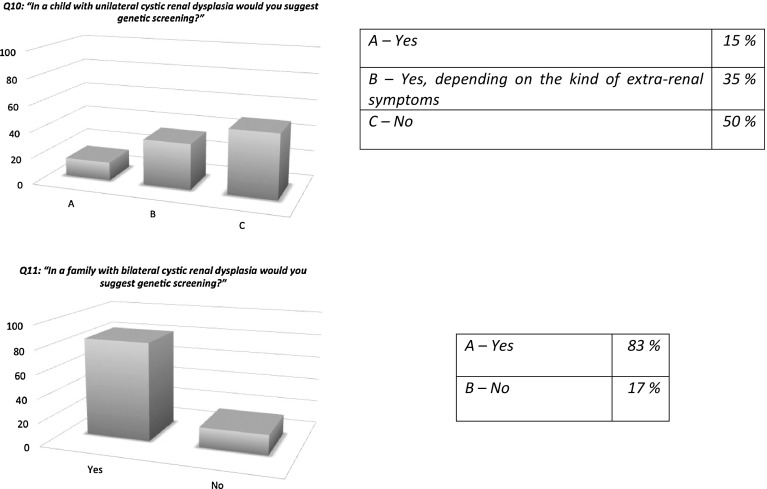
Genetics of renal dysplasia

**Fig. 4:**
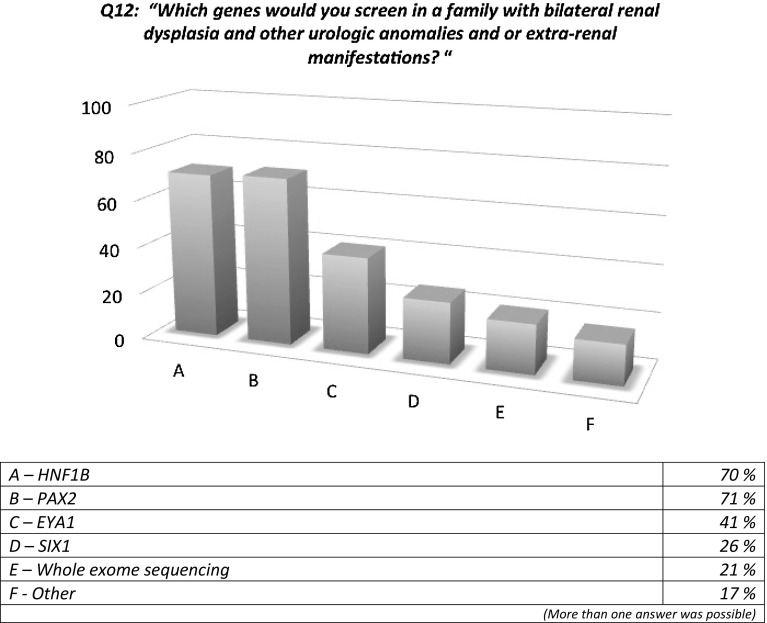
Genetics of renal dysplasia

Responders would be more inclined to perform genetic screening in children affected by bilateral RD (60%) rather than in those with unilateral RD (19%) (questions 8 and 9), and similar percentages were found for the cystic forms of RD, with a lesser tendency to perform further genetic testing in cases of unilateral RD when extra-renal manifestations were present (questions 10 and 11) (Fig. 3). The genes that responders would most frequently screen were *TCF2*/*HNF1B* and *PAX2* (70 and 71%, respectively) followed by *EYA1* and *SIX1*, while more than 20% of responders suggested whole exome sequencing (question 12) (Fig. 4).

## Discussion

The survey showed that there is a total lack of homogeneity among pediatric nephrologists as far as the diagnosis and management of children affected by RD are concerned.

### Diagnosis

The only diagnostic criterion reported in the literature is histological and not feasible in clinical practice. As regards clinical practice and also research, it is essential to have a universally accepted definition of renal dysplasia for more than one reason. Firstly, since dysplasia represents the most common cause of CKD in children, it is important to diagnose it correctly; secondly, when classifying these conditions in registries or when reporting on the progression of the disease or on results of interventional studies, it is vital that everybody starts from the same point, which is a diagnosis based on the same set of criteria.

Parenchymal hyperechogenicity, regardless of whether it is associated to a loss of cortico-medullary differentiation, is the only US feature that differentiates RD from simple renal hypoplasia and it is essential for a diagnosis of RD. Once other causes of increased renal echogenicity (such as congenital nephrotic syndrome, polycystic kidney disease, glycogen storage disease, nephronophthisis and oxalosis) have been excluded, parenchymal hyperechogenicity could suggest a diagnosis of RD in an infant or young child. This is an important indicator, as a kidney biopsy is neither clinically nor ethically indicated. Having said that, US alone cannot be considered the bedrock for a diagnosis of RD *per se*, as there has never been a study reported in the literature that correlates postnatal US or other radiology images with definitive histology of dysplasia. Instead, a clinical diagnosis of RD should be based on a whole suite of available clinical data. If antenatal scans are also abnormal, a diagnosis of RD is quite likely, but if they are normal, the diagnosis becomes less so. If antenatal scans are not available, then the following criteria must be carefully examined: the perinatal history (e.g. an abnormal kidney due to prematurity or renal vein thrombosis); the early childhood history (e.g. severe urosepsis may point to acquired kidney scarring in a small kidney, or the presence of severe hypertension may point to a small kidney with renal artery stenosis); the family history which may include RD (or things which may look similar on postnatal US such as nephronophthisis); and the use of genetic testing. When no hyperechogenicity shows up on US, then a diagnosis of RD cannot be made. When advanced CKD is present, a diagnosis of bilateral RD is impossible to confirm, because with the late stages of kidney damage the anatomical features are similar for a range of diseases. Small kidneys can be present in association with hyperechogenicity, consistent with renal hypo-dysplasia.

### Imaging

Due to the lack of guidelines and recommendations in the literature, imaging in children affected by RD is an open field of debate. Renal dysplasia is often associated with urinary tract abnormalities, most commonly vesicoureteral reflux (VUR) and posterior urethral valves [5], and can sometimes be a consequence of obstructive uropathy *in utero* [9, 10]. The ESPN panel believes that a more thoughtful and specific approach is needed in order to detect particularly correctable malformations of the urinary tract, especially obstructions, and manage and follow-up children with VUR in accordance with local guidelines. Therefore, in our clinical experience, further imaging, such as VCUG, DMSA scanning and indirect cystography with mercapto-acetyltriglycine (MAG3), could be beneficial only in children with bilateral or unilateral RD in association with clinical symptoms such as urinary tract infections or major dilations of the urinary tract, especially of the ureter.

Imaging investigation in families of children affected by RD is an intriguing aspect, and we agree that it would be beneficial to perform US screening in first degree relatives of children with non-obstructive bilateral RD—in other words, in those without abnormalities of the urinary tract—in order to detect unrecognized and asymptomatic cases. US screening of first degree relatives seems useful, as the economic cost is relatively low with no radiation burden, while the clinical usefulness of RD diagnosis is relevant in allowing for an ad-hoc follow-up. Furthermore, US screening of siblings and parents can be helpful in trying to identify specific hereditary patterns within affected families. Extended fetal renal ultrasound screening in new pregnancies of parents with affected children could also be of value.

### Genetics

The genetic aspect of RD is currently a hot topic in the literature. The significant improvements in genome scanning technologies, such as next generation sequencing, have enabled the study of large areas of DNA from affected children in an attempt to unravel the genetic background of this condition. Over the last few years, mutations in many genes have been associated with non-obstructive RD. Some of these are included in complex clinical syndromes such as branchio-oto-renal syndrome (*EYA1, SIX1*), renal-coloboma syndrome (*PAX2*), renal cysts and diabetes syndrome (*TCF2*/*HNF1B*) and Townes–Brocks syndrome (*SALL1*). However, the majority of RD cases are non-syndromic, oligogenic or polygenic disorders, which do not follow Mendelian inheritance and may occur sporadically [11, 12]. The numbers of genes involved in non-syndromic RD is constantly growing and particular attention has been focused on hepatocyte nuclear factor 1-beta (*HNF1B*) mutations, which have been described as the main cause of bilateral hyperechogenic kidneys at prenatal US [13]. *HNF1B* encodes a transcriptional factor involved in the development of several tissues, including that of the kidney and urinary tract, pancreas and others [14]. The most common pathological expressions of *HNF1B* mutations are renal cysts associated with early onset diabetes. Twenty-five percent of patients with bilateral cystic RD carry an *HNF1B* mutation [14]. Therefore, it seems reasonable to study mutations of *HNF1B* in these children, especially when cysts are found in association with hyperechogenicity. Major clinical implications from this genetic diagnosis derive from the fact that mutations in this gene may cause noninsulin-dependent diabetes mellitus later in life and that it is inherited as an autosomal dominant trait. Recently, a score system has been proposed to select patients that should be tested for HFN1B mutations, showing good results [15]. As there is a limited genotype-phenotype correlation, familial screening of *HNF1B* should be proposed when mutations have been identified in the index patient, in order to offer adequate genetic counselling [16]. However, given the contentious nature of screening siblings of affected children due to medical and psychological implications, caution is needed and genetic testing should only be offered to families deemed responsible enough to deal with the eventual results. The potential influence on life insurance policies is something that also needs to be taken into consideration.

A more extensive genetic screening (gene panel diagnostics or whole exome screening) could be useful in patients with severe syndromic disease with multiorgan manifestations or when a differential diagnosis is required, e.g. between nephronophthisis and renal hypo-dysplasia (RHD), which can be very hard to separate on clinical grounds, and/or for research purposes. In the near future, when gene panel testing becomes routinely available, a relatively large number of genes will be tested at low cost and this will change the scenario, allowing a more in-depth genetic testing in clinical practice.

## Limitations of the study

All the data reported and discussed derive from an on-line survey, addressed to European pediatric nephrologists, with no contribution being made by other professionals, who could be involved in the diagnosis and care of children with renal dysplasia. Furthermore, only 20% of the pediatric nephrologists contacted about the survey by mail responded to the survey, and therefore they represent a partial and not fully representative sample. Another limitation of the study relates to the fact that some of the questions dealing with the diagnostic process adopted by pediatric nephrologists following a diagnosis of renal dysplasia allowed for multiple answers, and this could have led to some difficulty in interpreting the responses.

## Conclusion

In summary, bearing in mind the above-mentioned limitations, this survey clearly unveils the heterogeneity of diagnostic measures and management of RD among specialists throughout Europe. As Pediatric Nephrologists and other specialists with expertise in the field, from different countries, we believe in the importance of establishing widely accepted general guidelines. This will improve the quality of care for children with RD who are at risk of developing chronic renal insufficiency and end-stage renal disease.
